# Is COVID-19 a Crime? A Criminological Perspective

**DOI:** 10.1007/978-3-030-65355-2_29

**Published:** 2021-03-20

**Authors:** Toine Spapens

**Affiliations:** 1 Tilburg University, Tilburg, The Netherlands; 2 Tilburg University, Tilburg, The Netherlands; 3 Tilburg University, Tilburg, The Netherlands; 4 Tilburg University, Tilburg, The Netherlands; Department of Criminal Law, Tilburg Law School, Tilburg, The Netherlands

## Abstract

Is COVID-19 a crime? The answer to that question seems relatively straightforward. Although the virus may be viewed as a “villain,” we cannot treat it as a criminal. However, how the virus impacts societies and government responses to the crisis raises serious criminological questions. In this chapter, I briefly address three. I will start by looking at the effects of COVID-19 and particularly the lockdowns on criminal activities. My second question is whether we should rethink our response to crimes that may facilitate future pandemics, particularly wildlife trafficking. Finally, I will discuss some examples of systemic inequalities, which affect the impact of the virus on societies. Given the current state of affairs, I will raise questions and ideas for future research, rather than provide clear-cut answers.

Is COVID-19 a crime? The answer to that question seems relatively straightforward. Although the virus may be viewed as a “villain,” we cannot treat it as a criminal. However, how the virus impacts societies and government responses to the crisis raises serious criminological questions. In this chapter, I briefly address three. I will start by looking at the effects of COVID-19 and particularly the lockdowns on criminal activities. My second question is whether we should rethink our response to crimes that may facilitate future pandemics, particularly wildlife trafficking. Finally, I will discuss some examples of systemic inequalities, which affect the impact of the virus on societies. Given the current state of affairs, I will raise questions and ideas for future research, rather than provide clear-cut answers.

## The Effects of COVID-19 on Criminal Activities

It is an interesting question how COVID-19 affected the behavior of criminals. At the beginning of the crisis, it was clear that some were quick to seize the opportunity to fraudulently offer products such as hand gels, facemasks, and other protective equipment of questionable quality, which were sometimes not delivered at all. However, as governments stepped up production, such opportunities rapidly diminished. The lockdowns imposed in many countries also had a noticeable effect on crime. For several weeks, figures on shoplifting, burglaries, and other street crimes decreased to almost zero. From a theoretical perspective, this is understood by applying the routine activities theory, which predicts that crime occurs when a motivated offender, a suitable target, and a lack of capable guardians converge in time and space (Cohen and Felson 1979). The lockdowns probably affected all parameters. There were fewer targets, guardianship increased, whereas convergence settings decreased. Furthermore, criminals are human too, and on average not very healthy (Odgers et al. 2007), and we may hypothesize that this caused them to avoid the risks of catching the virus. On the other hand, several countries such as the United Kingdom have reported a substantial increase in intimate partner violence (Townsend 2020). Here, unfortunately, the lockdowns resulted in “motivated offenders” and “suitable targets” being in each other’s vicinity more often than usual.

The effect of lockdowns on more complex organized crimes is less clear. Opportunism was visible in the shape of the Italian mafia offering loans to small entrepreneurs who were hit by a lack of customers, but essentially this type of “philanthropy” was probably built on the loan sharking activities the mafia has traditionally been involved in (D’Angelo and Musumeci 2016). So far, Dutch drug producers and traffickers seem hardly affected by COVID-19. Perhaps ecstasy is less in demand because of the canceled festival season and the ongoing restrictions on nightlife, but little has changed in the market for cocaine, according to preliminary enforcement agencies’ information (EMCDDA and Europol 2020). Governments’ efforts to continue international trade and transport meant business as usual for traffickers, judging from recent seizures of cocaine shipments in Dutch ports. Small-scale distribution to customers was not affected either particularly because Dutch criminals increasingly ship drugs in postal packages. Although means of production for synthetic drugs seem to be less easy to obtain, large synthetic drugs producers usually buy large amounts of precursor chemicals when on offer and stockpile what they do not need immediately (Spapens 2016; KLPD 2012) Assuming that they still hold reserves, short- term effects on production seem unlikely.

A question that has worried authorities is whether criminals who lost their illegal income have shifted to other “markets” and to cybercrime in particular. Here, opportunities are assumed to have increased because more people are working online from home. Indeed, the numbers of cyberattacks reported to the police rose, but the question remains whether this can be explained by increased efforts of existing cybercriminals or because others have switched to online crimes. Criminological research does show that those involved in complex crimes tend to specialize rather than multitask (Spapens 2017). Skills are more important than one might think, as are mindsets. Those involved in relatively simple property crimes are usually looking for easy money and immediate satisfaction of needs but often lack patience for crimes that require more time to render a profit (Spapens 2017).

If we may draw a general conclusion from the observations above, it is clear that the period in which measures were imposed on people’s freedom is yet too short to cause substantial changes in criminals’ behavior.

## Rethinking Our Response to Crimes That May Facilitate Future Pandemics, Particularly Wildlife Trafficking

Whilst epidemiologists have been warning us for the risk of a pandemic for many years, “green” criminologists have also expressed worries in connection to wildlife trafficking. Even if animal trade is legal and meets regulatory requirements, it brings live animals and animal products into close proximity with people engaged in commerce and consumption/use, whether as food, pets, medicinal ingredients, or for other purposes (Broad 2020). That is not to say that illegal activity presents no added risks. Poor transport conditions, avoidance of quarantine controls on import, or black-market trade outside regulated markets and retail outlets where health inspections may be focused certainly presents incremental concerns (Broad 2020).

In the past decades, illegal trade in endangered wildlife has developed into one of the most important types of environmental crime. The trade in live animals mostly concerns protected birds and reptiles. Although Asia and China, in particular, are the most important destinations for trafficked wildlife, demand for exotic animals remains high also in the United States and Europe (Van Uhm 2016). The Netherlands, for instance, is a major hub for the trade in exotic birds (Van Uhm and Spapens 2018).

Apart from its detrimental effects on endangered species’ survival as well as on local ecosystems, there is also a substantial risk that living animals carry viruses and diseases. However, until COVID-19, the problem was primarily perceived as economic because diseases could spread to production animals (Van Uhm and Spapens 2018). Countries apply extensive sanitary rules when it comes to wild animals, but traffickers logically circumvent health checks. The trade in wildlife is as such not illegal but regulated as a normal economic activity. Consequently, first-line enforcement falls upon administrative agencies, which usually have limited powers of investigation whereas maximum prison sentences and financial penalties are often low (Elliott 2009; Spapens 2014).

The question is, therefore, whether we should now drastically rethink our approach to wildlife trafficking. The trade in wildlife is currently regulated in the Convention on International Trade in Endangered Species of Wild Fauna and Flora (CITES). CITES focuses on protecting endangered species against overexploitation and the risk of extinction but does not specifically address the risk of diseases. Furthermore, it leaves many legal and practical loopholes that criminals may exploit. Of course, a purely repressive approach will not suffice: it is far more important to develop interventions that help to change our socially constructed appreciation of the value of scarce wildlife.

## Systemic Inequalities and the Impact of COVID-19

There is extensive literature on how crime and other problems affect vulnerable groups in particular, and COVID-19 is no exception. Critical or “radical” criminologists focus, on the one hand, on systemic drivers of such inequalities and, on the other hand, look at the roles of corporate and government actors. Contrary to mainstream criminologists, they have also argued that the object of study of criminology should not be limited to behavior included in penal codes. Indeed, a wide range of damaging behavior is not criminalized at all, for example, tax avoidance and unregulated fishing. Therefore, the focus should also be on “blameworthy harm,” structural inequalities, and on “crimes of the powerful,” particularly by corporate and governmental actors.

Relevance of societal inequalities in the context of COVID-19 can easily be illustrated. For example, countries such as the United States and the United Kingdom report far more casualties amongst ethnic minorities, particularly amongst socially and economically deprived groups (Price-Haywood et al. 2020; Barr et al. 2020). In the Netherlands, the effects of inequality are visible in the labor market. For example, the virus has disproportionally hit Eastern European migrant workers in the meat sector. Conditions at the workplace, housing, and transport increase risks, for example, because rules of social distancing cannot be met (Winkel 2020). Another worrying sign is that COVID-19 generally appears to have a heavier impact in areas where pollution problems are higher. In the Netherlands, this possibly applies to parts of North-Brabant and Limburg with a high-density bio-industry and the resulting environmental problems. Earlier, these areas were also struck more severely with Q-fever (Lucassen 2020). Although establishing specific causal effects would require extensive research, consensus is that where large numbers of humans and non-human animals—either wildlife or production animals—live in close proximity, the risk increases that zoonotic diseases develop and spread (Broad 2020).

From a criminological point of view, the efforts of governments to reduce the harmful impact of COVID-19 on societies are equally important. This encompasses a wide range of issues, which I can mention only briefly. To begin with, negligence may be considered as causing blameworthy harm. It may also offer criminals opportunities to step into the vacuum left by legitimate authorities, for example, in Rio de Janeiro’s favelas where drug gangs imposed a lockdown to protect inhabitants since President Bolsonaro did not take the virus seriously (Schipani and Harris 2020). Second, it raises classic questions. To what extent are governments allowed, for instance, to rein in civil liberties to prevent harm by applying instruments of mass surveillance or otherwise? What is the lesser harm: people dying of COVID-19 or economic damage?

Whether COVID-19 will be a flashpoint leading to shifts in criminal markets; rethinking how we relate to non-human animals and wildlife, or even restructuring some of the structural flaws of the “treadmill of production” that characterizes our dominant economic system and shapes our societies remains an open question.
